# Social exclusion in people with diabetes: cross-sectional and longitudinal results from the German Ageing Survey (DEAS)

**DOI:** 10.1038/s41598-023-33884-8

**Published:** 2023-05-02

**Authors:** Tino Prell, Stefanie Stegmann, Aline Schönenberg

**Affiliations:** Department of Geriatrics, Halle University Hospital, Halle, Germany

**Keywords:** Diabetes, Quality of life

## Abstract

As social exclusion can be linked to worse health and overall reduced quality of life, we describe social exclusion in people with diabetes and assess whether diabetes can be considered as a risk factor for social exclusion. We analyzed two waves (2014, 2017, N = 6604) from a survey of community-dwelling people aged > 40 using linear regression, group comparison and generalized estimating equations to explore the association between diabetes, social exclusion, socioeconomic, physical and psychosocial variables. In the entire cohort, diabetes was cross-sectionally associated with social exclusion after adjusting for covariates (*p* = 0.001). In people with diabetes, social exclusion was further associated with self-esteem (*p* < 0.001), loneliness (*p* =  < 0.001), income (*p* = 0.017), depression (*p* = 0.001), physical diseases (*p* = 0.04), and network size (*p* = 0.043). Longitudinal data revealed that higher levels of social exclusion were already present before the diagnosis of diabetes, and future social exclusion was predicted by self-esteem, loneliness, depression, and income, but not by diabetes (*p* = .221). We conclude that diabetes is not a driver of social exclusion. Instead, both seem to co-occur as a consequence of health-related and psychosocial variables.

## Introduction

Under the slogan “Leave No One Behind”, The United Nations (UN) made social inclusion the main goal of their 2030 Agenda for Sustainable Development^1^. Social *exclusion* describes a state in which people are unable to participate fully in economic, social, political, and cultural life.^1^ Due to the highly complex and continuously unfolding nature of the phenomenon, the definitions of social exclusion are imprecise and vague.^1–3^ Social exclusion can occur in many ways and on different levels, and manifests ultimately in a lack of rights or political representation, as well as limited resources, for example on a financial, educational, nutritional, or health-related level. These shortcomings often cumulate in reduced social participation, quality of life, and health^1,3–5^. A Japanese study on older adults even links social exclusion to premature death^6^. Importantly, social exclusion cannot be understood as a purely objectivist concept^7^. The interrelated objective dimensions of exclusion, i.e. socioeconomic marginalization and social isolation, are the basis for the resulting feelings of not being included in society. This means that the subjective perception of one's own situation plays a decisive role in the feeling of exclusion^8^. Of note, although they often co-occur, social exclusion and social isolation are not interchangeable terms. While social isolation describes social connectedness and refers to the actual contact an individual has with family, peers, neighbors or society as a whole^9^, social exclusion describes dimensions that go further, including access to basic healthcare, infrastructure, education, and political representation^1^. Another closely related concept is stigmatization. Stigma is closely linked to and can enhance social exclusion, as stigmatized persons are systematically excluded from resources and opportunities such as education, housing, employment, and health and social care.^10–12^.

Much like the manifestations of social exclusion, its reasons are manifold^1,2,12^. Common reasons for social exclusion are age or sex, ethnicity, religion, or economic status^10,13^. Additionally, disability or health problems often lead to exclusion^1,5^. From a medical perspective, social exclusion often manifests in reduced access to basic healthcare such as medication, preventive measures and regular doctor consultation, or hospital stays for marginalized groups.^14^ Therefore, social exclusion not only impacts mental health due to the feeling of being separate from society,^15^ but also physical health due to inability to perform daily activities, and reduced access to healthcare for financial, infrastructural, and informative reasons^5,16–19^. In addition to social signals of exclusion, such as verbal discrimination or criminality, for people with health problems, the basic infrastructure failings (e.g., buildings without elevators, no public transport) can diminish the participation in public life. Consequently, in a survey among 860 Dutch households, a causal analysis showed that having bad health is the most important risk factor for social exclusion^20^. Likewise, a longitudinal UK-based survey found a bidirectional relationship between poor health and social exclusion^5^, and an Irish study affirms that physical disability is associated with higher levels of social exclusion.^21^.

According to the world health organization (WHO), diabetes was on place nine of the leading causes for death worldwide in 2020, and its prevalence is increasing further^13^. Besides the management of the physical and medical aspects of diabetes, there has been increased research interest in the psychosocial aspects of this chronic disorder. Living with diabetes can be associated with stigma, which impairs quality of life and increases diabetes-specific distress and complications due to lack of self-care^10,22–25^. This stigmatization can come both from other people as well as from patients themselves^10^, and people with diabetes may generally show reduced self-esteem^26^. Previous studies have also shown a link between diabetes and loneliness^27,28^, with a 20-year follow up study from Sweden confirming that loneliness increases the risk for developing type 2 diabetes.^29^ Likewise, social support plays an increased role for people with diabetes^30^, for example, a Dutch study links social isolation, social network and work-related provisions with diabetes type 2, which the authors attribute to symptoms such as tiredness or general feelings of being unwell.^31^ Diabetes is furthermore linked to multimorbidity, reduced participation in daily activities, and physical as well as mental health problems^13,32^. All these constructs are closely linked to social exclusion as a palpable, society-wide derogation, therefore, it is of interest to examine whether this common illness is also related to social exclusion directly.

So far, the association between diabetes and social exclusion on society level has not been studied in detail, despite its potential impact on mental and physical health. Thus, based on a nationally representative sample of German adults, we aimed to explore social exclusion in people with diabetes. First, we compared social exclusion between individuals with and without diabetes to assess whether social exclusion was pronounced in people with diabetes. In a second step, we aimed to understand the particular characteristics of social exclusion in people with diabetes. For this purpose, we determined how social exclusion in people with diabetes is associated with various medical and psychosocial cofactors that are known to be relevant for social exclusion^10,11,22^: sex, higher BMI/obesity^10,33,34^, socioeconomic status^35^, multimorbidity^36^, depression^10,22,23^, loneliness^28^, self-esteem and self-efficacy^22,23^, physical function^10^, and autonomy^37^. Finally, we used longitudinal data to explore the onset of social exclusion in people with diabetes, and whether diabetes itself can be considered a risk factor for social exclusion.

## Methods

### Sample

The data were taken from the public release of the German Ageing Survey (Deutscher Alterssurvey, DEAS), conducted and provided by the Research Data Centre of the German Centre of Gerontology (Deutsches Zentrum für Altersfragen, DZA) and funded by the Federal Ministry for Family Affairs, Senior Citizens, Women and Youth. The DEAS is a representative cross-sectional and longitudinal survey of the community-dwelling population aged 40 and above in Germany, with the main goal of assessing physical and mental health, living conditions, psychosocial parameters and well-being in middle-aged and older adults^38^. For this purpose, representative population samples were drawn at each wave (cross-sectional data) and participants completed a computer-assisted interview (CAPI) as well as a drop-off self-report questionnaire. Participants of each wave were also invited to complete future waves, leading to a rich longitudinal data register. Therefore, it is well-suited to provide data about social and medical determinants of well-being and provides a multitude of variables relevant to the presented research questions. The DEAS is an ongoing survey with currently six waves (1996–2017) and several shorter surveys during the COVID-19 pandemic (2020–2022)^37^. In accordance with the German Research Foundation (Deutsche Forschungsgemeinschaft, DFG) and the Institute for Applied Social Science (INFAS), no ethics approval was needed for the study as data were collected under pseudonyms and voluntarily, and the study was deemed low-risk, as no work on patients was included. Nonetheless, both institutions approved the study, and data collection was conducted according to the Declaration of Helsinki. Written informed consent was obtained from all participants. For more information, please refer to the homepage of the DZA (https://www.dza.de/en/research/fdz/german-ageing-survey) and the related publications^38–41^. As the questionnaires and variables included in the survey vary between waves (social isolation, for example, was only added to the survey instrument list in 2014), we selected the most recent two waves that contained all the relevant variables (see below). We refrained from using the latest waves due to the potential bias introduced to the data collected during the COVID-19 pandemic, which strongly influenced physical and mental health. Therefore, we used cross-sectional and longitudinal data from the fifth (2014) and sixth wave (2017) (see section Data Availability). The study population included people with and without diabetes in both waves.

### Variables

In the CAPI, the presence of diabetes was assessed via self-report (yes/no); participants were asked if they had ever been told by a doctor that they suffered from diabetes – of note, the question did not differentiate between type 1 and type 2 diabetes.

Social exclusion was assessed using the scale by Bude and Lantermann^7^. It consists of four items ranging from 1 = “strongly agree” to 4 = “strongly disagree”: “I am worried to be left behind”, “I feel like I do not really belong to society”, “I feel that I am left out”, and “I feel excluded from society”. In line with the DEAS guidelines, the sum score of the scale was treated as a continuous variable^42^, with higher values representing higher perceived social exclusion.

In addition, the following sociodemographic, psychosocial, and medical covariates were extracted from the database:

- Socioeconomic: age, gender, marital status, monthly net equivalent income (OECD scale), education level based on the International Standard Classification of Education (ISCED) resulting in the levels low (ISCED 0–2), medium (ISCED 3–4) and high (ISCED 5–6)
- Self-reported presence of diabetes (yes/no)
- BMI, 2017
- SF36 Short form health survey, 2017^43^
- Total number of physical diseases, 2017. This variable is based on a self-report during the CAPI. Participants were asked to select which diseases they had been diagnosed with by a doctor from a list (1 = yes/0 = no)
- Number of important people in regular contact, 2017
- Depression Scale ADS, 2017 (German short version of CES-D-Scale^44^)
- 6-Item Scale for Loneliness, 2017^45^
- Satisfaction With Life 2017^46^
- Generalized Self-Efficacy Scale, 2017^47^
- Self Esteem Scale, 2017^48^
- German Perceived Autonomy in Older Age Scale, (WAA), 2017^49^

### Statistical analysis

All analyses were conducted using IBM SPSS statistics (Version 25), JASP (Version 0.16), and R (Version 4.1.1). The statistical significance was determined with *p* < 0.05. Missing values were treated with pairwise deletion. All cross-sectional analyses were performed based on the sixth wave (2017).

First, descriptive statistics were used to characterize the sample. Normality was assessed with the Shapiro–Wilk test, revealing non-normal distributions (*p* < 0.001) for all variables. Univariate group comparisons via Mann Whitney U test or Chi^2^ test were performed to determine differences between people with and without diabetes. Second, multiple linear regressions were used to analyze the association between social exclusion (dependent variable) and the above mentioned covariates (independent variables) using a stepwise selection algorithm and the AIC as selection criterion. Multicollinearity was assessed using the variance inflation criterion (VIF), revealing values between 1.07 and 2.20 at most. VIF values between 1 and 5 can be considered low to moderate; as it is common practice to remove variables with a VIF ≥ 5, no variables were removed in our models^50^. Finally, the dynamics of social exclusion were studied between the wave 2014 and 2017 by using paired Wilcoxon test and Generalized Estimating Equations (GEE) to account for repeated measures and within-person design.

## Results

### Factors associated with social exclusion

**Figure 1:**
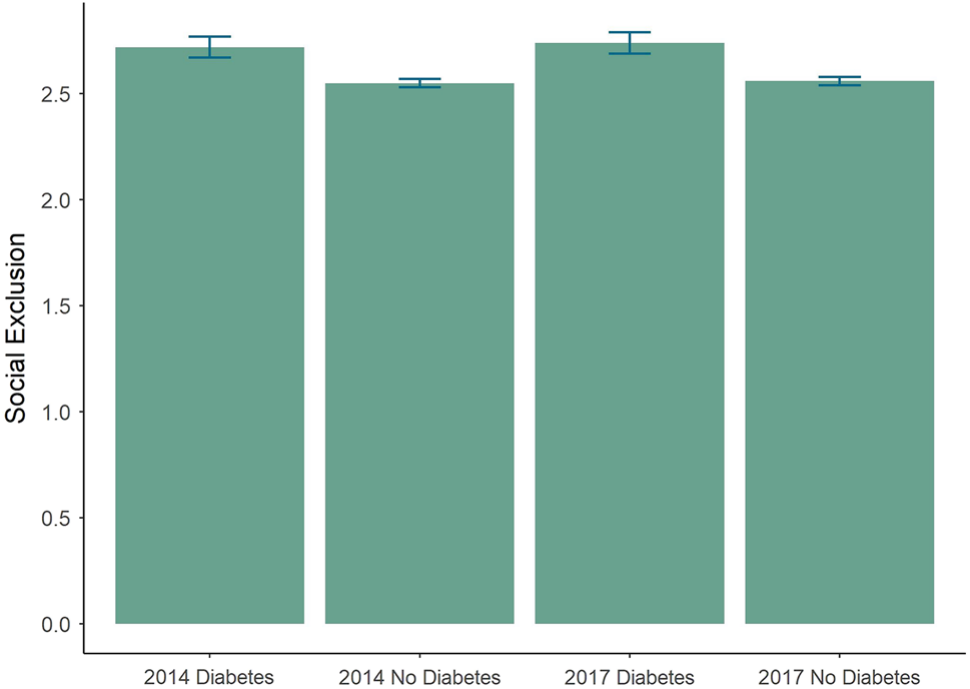
Mean Social Exclusion for participants with and without diabetes in 2014 and 2017 with 95% confidence intervals.

As a first step, we aimed to understand how participants with diabetes differed from those without diabetes in terms of health, well-being and social exclusion. Of the N = 6604 participants in wave six (2017), 13.5% (N = 897) reported to have been diagnosed diabetes mellitus (mean age 70.7 ± 9.5 years, 42.6% female). Detailed sample characteristics of the respective participants are given in Table 1. Group comparisons between participants with and without diabetes revealed higher levels of social exclusion in participants with diabetes in both waves (Fig. 1**)**, with small-to-medium effect size (*r* = -0.153 in 2017 and *r* = -0.127 in 2014). Likewise, people with diabetes were older (*r* = -0.257), had a higher BMI (*r* = -0.372), a higher number of physical diseases (*r* = -0.477) and worse physical functioning according to the SF36 (*r* = 0.336). Additionally, people with diabetes had a lower monthly income (*r* = 0.217), lower self-esteem (*r* = 0.136) and autonomy (*r* = 0.109), and more depressive symptoms (*r* = -0.154) (see Table 1).

**Table 1:** Characteristics of the entire cohort (wave 2017).

Variable	Entire cohort	Diabetes	No diabetes	Group comparison*	
	N = 6604 N (%)	N = 897 (13.6% ) N (%)	N = 5707 (86.4%) N (%)	p*	Effect size
Sex: Male Sex: Female	3336 (50.3)	487 (57.4)	2667 (49.4)	< 0.001	0.055
	3290 (49.7)	362 (42.6)	2773 (50.6)		
Education: Low (ISCED 0–2) Education: Medium (ISCED 3–4) Education: High (ISCED 5–6)	340 (5.4)	62 (7.3)	273 (5.1)	< 0.001	0.058
	3138 (50.1)	466 (54.9)	2664 (49.3)		
	2787 (44.5)	321 (37.8)	2463 (45.6)		

	M (SD)	M (SD)	M (SD)		
Social exclusion	2.59 (.58)	2.74 (.65)	2.56 (.57)	< 0.001	− 0.153
Age (years)	66.61 (10.93)	70.73 (9.49)	65,86 (10.98)	< 0.001	− 0.257
Social network size (number of important people in regular contact)	5.08 (2.78)	4.75 (2.73)	5.13 (2.76)	< 0.001	0.076
Body-mass-index (BMI)	27.03 (4.69)	29.68 (5.15)	26.61 (4.49)	<0 .001	− 0.372
SF36 physical functioning (standardized)	80.81 (23.79)	68.82 (28.56)	83.01 (21.88)	< 0.001	0.336
Total number of physical diseases	2.67 (2.01)	4.18 (2.13)	2.42 (1.89)	< 0.001	− 0.477
Depression scale ADS/ German short version of CES-D-Scale	6.69 (6.00)	8.00 (6.42)	6.46 (5.87)	< 0.001	− 0.154
6-Item scale for loneliness	1.75 (.54)	1.82 (.54)	1.74 (.54)	0.001	− 0.067
Satisfaction with life	3.85 (.70)	3.75 (.44)	3.87 (.69)	<0 .001	0.081
Generalized self-efficacy scale	3.08 (.43)	3.03 (.44)	3.08 (.43)	< 0.001	0.090
Self esteem scale	3.41 (.40)	3.33 (.40)	3.42 (.40)	< 0.001	0.136
Autonomy in older age	4.49 (.52)	4.39 (.54)	4.50 (.51)	< 0.001	0.109
Monthly equivalence income (in EUR, new OECD equivalence scale)	2,076.14 (1,318.18)	1,779.52 (1,170.77)	2,129.82 (1,344.57)	<0 .001	0.217

*Group comparison performed for people with and without diabetes. P-values based on Mann–Whitney U test for metric and chi^2^ test for categorical variables. Effect size for categorical variables = Cramer´s V, Effect size for metric variables = rank biserial correlation. Effect size less than 0.3 indicate a small, between 0.3 and 0.5 a medium, and effect sizes greater than 0.5 a large effect^50^.

*M* Mean, *SD* Standard Deviation.

When using stepwise regression models to assess social exclusion in the entire cohort (N = 6604), the diagnosis of diabetes was independently associated with social exclusion (*p* = 0.001) after adjusting for psychosocial and sociodemographic covariates (Table 2a). Therefore, we proceeded to assess the predictors of social exclusion in the entire study population as well as in people with diabetes in particular.

**Table 2:** Linear regression for social exclusion (wave 2017) in A) the entire cohort and B) people with diabetes.

	Coefficient	*P*	Beta
**Model A: entire cohort (N = 6604)**^a^			
Constant	4.978	< 0.001	
Self Esteem	− 0.606	< 0.001	0.664
Loneliness	0.226	< 0.001	0.201
Monthly income (in EUR)	− 0.0001	< 0.001	0.036
Sex (male)	− 0.066	< 0.001	0.023
Intra− familiar relationship (very good)	− 0.086	0.007	0.013
Intra− familiar relationship (good)	− 0.082	0.008	0.013
Intra− familiar relationship (moderate)	− 0.069	0.036	0.013
Intra− familiar relationship (poor)	0.073	0.215	0.013
Depression	0.005	< 0.001	0.012
Satisfaction with life	− 0.039	0.001	0.009
Number of physical diseases	0.013	0.001	0.009
Diabetes	− 0.061	0.001	0.009
Autonomy	− 0.038	0.004	0.007
**Model B: people with diabetes (N = 897)**^b^			
Constant	4.603	< 0.001	
Self esteem	− 0.557	< 0.001	0.520
Loneliness	0.278	< 0.001	0.281
Depression	0.012	0.001	0.068
Monthly income (in EUR)	− 0.0001	0.017	0.036
Number of physical diseases	0.019	0.040	0.027
Social network size	− 0.013	0.043	0.026
Satisfaction With Life	− 0.066	0.047	0.025
Autonomy	− 0.058	0.116	0.016

^a^Stepwise selection, AIC. Adjusted R^2^ = 0.43. Entered independent variables: age, sex, social network size, Body-Mass-Index (BMI), SF36 Physical functioning (standardized), Education, Total number of physical diseases, Depression Scale ADS, 6-Item Scale for Loneliness, Satisfaction With Life, Generalized Self-Efficacy Scale, Self Esteem Scale, Scale noticed autonomy in older age, Monthly equivalence income, Intra-familiar relationship, diabetes (yes/no). Data from the entire cohort (N = 6604) from wave 2017.

^b^Stepwise selection, AIC. Adjusted R^2^ = 0.42. Entered independent variables: age, sex, social network size, Body-Mass-Index (BMI), SF36 Physical functioning (standardized), Total number of physical diseases, Depression Scale ADS, 6-Item Scale for Loneliness, Satisfaction With Life, Generalized Self-Efficacy Scale, Self Esteem Scale, Scale noticed autonomy in older age, Monthly equivalence income, Intra-familiar relationship, Education. Data from people with diabetes (N = 897) from wave 2017.

The strongest predictor of social exclusion was self-esteem, both in the entire cohort (*p* < 0.001, Table 2a) and when repeating the regression analysis in people with diabetes (*p* < 0.001, Table 2b). Both in people with and without diabetes, social exclusion was additionally linked to loneliness (*p* < 0.001), income (*p* < 0.001 in the entire cohort and *p* = 0.017 in the diabetes cohort), depression (*p* < 0.001 and *p* = 0.001), number of physical diseases (*p* = 0.01 and *p* = 0.040), and overall satisfaction with life (*p* = 0.001 and *p* = 0.047).

### When does social exclusion occur?

To understand how social exclusion and diabetes in particular are linked, we used longitudinal data to assess whether newly diagnosed diabetes serves as a predictor of subsequent social exclusion. Overall, 6265 persons were interviewed both in wave five (2014) and six (2017). From 2014 to 2017, 188 people reported new onset of diabetes. Group comparison of baseline parameters in 2014 among 1) people who were newly diagnosed with diabetes between 2014 and 2017, 2) people who already reported diabetes in 2014, and 3) people who did not develop diabetes is given in Table 3a. Of note, the level of social exclusion in people who were newly diagnosed with diabetes in 2017 differed significantly from those who did not report new-onset diabetes, but not from those who already reported diabetes in 2014, indicating that a higher level of social exclusion was already present before the diagnosis of diabetes. People who did not have diabetes in 2014 but developed diabetes by 2017 were already characterized by more chronic disorders, higher BMI, higher levels of both depression and loneliness, lower income, and poorer self-esteem in 2014 (Table 3a). To confirm this pattern, we performed a GEE on the variable social exclusion in wave six (2017) using variables from wave 2014 and 2017. Here, like in the previous models, future social exclusion was mainly predicted by self-esteem, loneliness, depression, and income (Table 3b) but not by diabetes itself (*p* = 0.221, 95% CI [-0.20–0.86]).

**Table 3:** Prediction of future social exclusion A) group comparison of people with diabetes in 2014, people who develop diabetes by 2017, and people who do not develop diabetes and B) GEE for social exclusion in 2017.

Variable	Do not develop diabetes by 2017		Develop diabetes by 2017		Diabetes in 2014	
	N	%	N	%	N	%
A: Group comparison based on paired Wilcoxon						
Sex: Male	2673	42.7	101	1.6	386	6.2
Sex: Female	2743	43.8	87	1.4	275	4.4

	M	SD	M	SD	M	SD
A: Group comparison based on paired Wilcoxon						
Social exclusion	2.55_b_	0.56	2.72_a_	0.71	2.73_a_	0.63
Age (years)	62.90_a_	11.00	64.38_a_	9.59	68.67_b_	9.25
Social network size	5.32_b_	2.71	4.65_a_	2.78	5.16_a.b_	2.70
(BMI	26.46_b_	4.26	29.85_a_	5.19	29.96_a_	5.15
SF36 Physical functioning	85.28_c_	19.87	76.91_a_	23.63	71.76_b_	26.40
Number of physical diseases	2.29_c_	1.68	3.23_a_	1.92	4.15_b_	1.89
Depression	6.25_b_	5.80	7.25_a.b_	6.20	7.80_a_	6.25
Loneliness	1.75_b_	0.53	1.88_a_	0.52	1.81_a.b_	0.58
Satisfaction with life	3.85_b_	0.70	3.69_a_	0.81	3.75_a_	0.78
Self-efficacy	3.09_a_	0.42	3.03_a_	0.49	3.05_a_	0.43
Self-esteem	3.42_b_	0.39	3.30_a_	0.44	3.34_a_	0.42
Autonomy	4.52_b_	0.49	4.45_a.b_	0.51	4.45_a_	0.54
Monthly income	2035.75_b_	1333.84	1678.70_a_	938.35	1732.28_a_	1612.53

	Estimate	95% CI	p			
B: Generalized estimating equations on social exclusion (Wave 2017), N = 1163						
Constant	10.34	8.35–12.34	** < .001**			
Diabetes	0.33	– 0.20–0.86	0.221			
Depression	0.02	0.00–0.03	**0.027**			
Age	0.00	– 0.01–0.02	0.742			
Gender (Male)	− 0.033	– 0.066–0.00	0.051			
BMI	0.00	0.00–0.00	0.452			
SF– 36 Physical scale	– 0.02	– 0.07–0.03	0.384			
Social network size	– 0.04	– 0.10–0.03	0.260			
Number of physical diseases	0.03	– 0.08–0.14	0.593			
Loneliness	0.08	0.05–0.11	** < 0.001**			
Satisfaction with life	– 0.03	– 0.06–0.00	**0.042**			
Self– efficacy	0.02	– 0.03–0.07	0.372			
Self– esteem	– 0.08	– 0.10–0.06	** < 0.001**			
Autonomy	– 0.09	– 0.15–0.02	**0.006**			
Monthly income	– 0.00	0.00–0.00	** < .001**			
Relationship with family	0.12	– 0.08–0.31	0.239			

^ab^Values in the same row and sub-table where the subscript is not identical differ greatly at *p* <0 .05.

*BMI* Body Mass Index, SF-36 = Short Form 36 Physical Functioning Subscale, CI = 95% confidence interval, significant predictors in bold.

## Discussion

In a representative study population of middle-aged and older German adults, we used group comparisons, linear regression and GEE to assess how diabetes and social exclusion are linked.

While social exclusion is increased in people with diabetes, our results indicate that diabetes itself only has a weak direct effect on social exclusion. Instead, social exclusion in people with diabetes is mainly driven by psychosocial aspects such as self-esteem, depression, social network size, and socioeconomic parameters. This is in line with our longitudinal observation that social exclusion precedes the onset of diabetes. These results indicate that the presence of diabetes itself is not necessarily an independent driver of social exclusion; instead, social exclusion in diabetes can be interpreted as a consequence of several other health-related and psychosocial conditions that occur in people with diabetes. Our study therefore extends the understanding of the association between diabetes and social exclusion and the moderating effects of the following various psychosocial factors^10,11^. These findings will be discussed in detail below.

We found that the strongest effect between diabetes and social exclusion was evident for self-esteem. In line with an earlier study^26^, people with diabetes reported lower self-esteem than people without diabetes. Self-esteem is the degree to which people have a favorable or unfavorable opinion of themselves and is significantly related to both mental and physical health^51^. It is considered an important psychological factor, even influencing glucose level and eventually the course of diabetes via psycho-neuroendocrine mechanisms or through stress-related unhealthy behavior^26^. This strong effect of self-esteem ties in with the results by Kato et al. 2020^23^ and Browne et al. (2013)^52^ on stigma, which report the perception of being responsible for having diabetes and considering the diagnosis a personal failure as the most prevalent aspects of stigma in diabetes. Our data underline the importance of self-esteem for well-being and social activity in people with diabetes, however, further studies are necessary to determine which aspects of self-esteem are particularly relevant. Moreover, cultural aspects of body-related self-esteem have to be taken into account when clinicians want to encourage positive body image because of its potential health benefits^53^. Interestingly, the BMI was not a significant predictor of social exclusion. This suggests that lower self-esteem cannot be reduced to being overweight or obese in the cohort of people with diabetes. It also shows that not obesity per se, rather than the own view towards the weight, is relevant for exclusion^54^. Additionally, as people with diabetes reported significantly more physical illnesses, the higher BMI may also be a byproduct of worse physical health and thus lack of activity, suggesting that it is not the body image but rather the lack of physical ability to participate that drives social exclusion.

The second most important predictor of social exclusion in people with diabetes was loneliness. Patients with diabetes frequently experience moderate loneliness^28^. As in our study, loneliness in people with diabetes was found to be associated with the presence of chronic disorders and younger age. At this point, it is worth noting that social exclusion and loneliness are distinct concepts. Perceived social exclusion describes the feeling that one does not belong to the society, whereas loneliness is the state that an individual’s social network is smaller than desired or the resulting support is lower than expected^42^. Loneliness is an emerging issue that is associated with deleterious outcomes and poor health^55^. For example, higher levels of loneliness were associated with subsequent higher levels of functional limitations, and higher levels of functional limitations were in turn associated with subsequent higher levels of loneliness, suggesting that the association between loneliness and functional limitations among people with diabetes is bidirectional^56^. Loneliness is a complex and multidimensional phenomenon, and the utilized De Jong Gierveld scale^45^ in the studied dataset is based on a multidimensional perspective containing overall, emotional, and social loneliness^45^.

The third most important predictor of social exclusion in our analysis was depression. In people with diabetes, we found higher levels of depressiveness in comparison to people without diabetes, although the effect size was small. In general, people with diabetes and especially those with obesity and physical inactivity, have an increased risk for depression^57^. Overall, lack of self-esteem, loneliness and poorer physical health are often associated with depression^58^.

In addition to self-esteem, loneliness, and depression, also the income, the number of chronic disorders, the social network size, life satisfaction, and autonomy were found to be associated with social exclusion in our analysis as well as in previous studies on stigma^4,12^. The current study suggests that the social network size mediates the effect on the relationship between diabetes and social exclusion. A large social network was associated with better intra-familiar relationships. One can therefore assume that social support and familial support are critical factors in overcoming social exclusion, as they may buffer the lack of acceptance from society in general^59^. The same can be assumed for a higher income.

Overall, our results show that social exclusion takes place in people with diabetes, and highlight the need to provide psychosocial support to people with diabetes in particular.

## Limitations

Our study is not free of limitations. Social aspects depend on cultural and economic characteristics within a society^11^ and may differ from country to country. This limits the generalizability of our results and highlights the need for further studies to take cultural aspects into account. Especially in Germany, a universal welfare state that offers insurance and financial support to all citizens, results regarding social exclusion may differ from other countries where such welfare structures are not in place^60^. Still, even in a developed welfare state, there are significant differences in social exclusion, health, and income for people with and without diabetes, but depending on the country and the supportive infrastructures, the predictors of social inclusion may vary. These country-specific limitations also hold for the practical implications of the presented results, as measures to reduce social exclusion depend on the structures already in place (e.g. financial support, caregivers, reduction of stigma in society, insurance, availability of public transport and accessible buildings for handicapped persons).

In addition, different measures for social exclusion exist and may partly explain the mixed findings, as the Bude and Lantermann^7^ tool, which was used here, covers only *perceived* social isolation. Generally, the provided data is based on self-report, which is always subject to bias, such as recall-bias and social desirability^61,62^. However, all instruments used in the surveys are validated and frequently used in the scientific literature, and self-report is required to assess subjective constructs such as life satisfaction, depressive symptomology, or feeling excluded ^1,8^. In addition, the use of a nationwide survey limits generalizability of the results, as a potential selection bias cannot be excluded. It is likely that people in nursing homes or hospitals, who may suffer from much more severe levels of both diabetes and social exclusion, are underrepresented in this dataset. Likewise, in the provided dataset, there was no differentiation between type 1 and type 2 diabetes, merely the overall diagnosis of diabetes was assessed. Again, self-report of diabetes may be critical, especially if blood-glucose levels are stable (e.g. due to medication) and people do not ‘feel’ that they have diabetes. To counteract this risk, the survey explicitly asks "Has a doctor ever told you that you are suffering from [Diabetes]”^39^. Likewise, many studies show that self-report of diabetes as used in survey data is reliable, especially when searching for social implications^63^ rather than medical aspects of the disease, where a more detailed assessment may be necessary^64–66^. Still, in future studies, it may be fruitful to differentially assess the relevance of social exclusion for the two types of diabetes. As diabetes type 1 often starts earlier in life, its dynamics may be different from type 2, for example due to the use of insulin^67,68^, although people with type 2 diabetes also report social distress^69^. Additionally, due to the nature of the dataset, the current occupational status was not properly represented. Based on the mean age of the participants, it is likely that most participants were retired. Still, as people without diabetes were younger than those with diabetes, it is possible that the occupational status may differ here. To incorporate the role of occupation for social exclusion, we included both education and monthly net income in our analyses, however, these variables cannot fully capture the psychological differences between work and retirement in terms of social roles, network, and daily life structure. Therefore, in future research, it would be of interest to differentially assess the relationship between social exclusion and occupational status, as it has been shown that occupational status influences social network size and well-being, especially mental health^70–73^.

Although the current analysis is strengthened by the large sample size and longitudinal data collection, it remains an exploratory overview to initially assess whether diabetes and social exclusion are linked at all and which variables contribute to social exclusion in this particular patient population. In future studies, it would be beneficial to understand how exactly diabetes and social exclusion are linked by using structural equation modelling or mediation analysis. This approach may shed further light into the direction of effects, especially concerning the relationship between physical heath/multimorbidity, self-esteem, social exclusion, and diabetes. This allows a deeper insight into the parameters linking social exclusion in diabetes, however, a stronger theoretical foundation of the important variables is necessary first.

## Conclusion

Social exclusion is relevant in people with diabetes, however, the illness alone is not a predictor of social exclusion. Instead, social exclusion in people with diabetes is mainly driven by psychosocial and health-related factors that are connected to the illness, which explains the cross-sectional association between both. Longitudinal results shed a light on the occurrence of social exclusion before the diagnosis of diabetes, showcasing that health-related and psychosocial factors may be the common cause linking both diabetes and social exclusion. Our findings have important implications for the understanding and improvement of social exclusion in people with diabetes and highlight the need to provide psychosocial support.

## Data Availability

The data are taken from the public release of the German Ageing Survey (DEAS), provided by the Research Data Centre of the German Centre of Gerontology (DZA). Data for wave 2014 10. 5156/DEAS.2014.D.001 and 2017 10. 5156/DEAS.2017.D.001 are freely available for scientific use after an initial registration from the homepage of the DZA (https://www.dza.de/en/research/fdz/german-ageing-survey).
